# Morphologic complexity of epithelial architecture for predicting invasive breast cancer survival

**DOI:** 10.1186/1479-5876-8-140

**Published:** 2010-12-31

**Authors:** Mauro Tambasco, Misha Eliasziw, Anthony M Magliocco

**Affiliations:** 1 Department of Oncology, University of Calgary, Calgary, Canada; 2 Tom Baker Cancer Centre, Calgary, Canada; 3 Department of Physics & Astronomy, University of Calgary, Calgary, Canada; 4 Department of Community Health Science, University of Calgary, Calgary, Canada; 5 Department of Pathology & Laboratory Medicine, University of Calgary, Calgary, Canada

## Abstract

**Background:**

Precise criteria for optimal patient selection for adjuvant chemotherapy remain controversial and include subjective components such as tumour morphometry (pathological grade). There is a need to replace subjective criteria with objective measurements to improve risk assessment and therapeutic decisions. We assessed the prognostic value of fractal dimension (an objective measure of morphologic complexity) for invasive ductal carcinoma of the breast.

**Methods:**

We applied fractal analysis to pan-cytokeratin stained tissue microarray (TMA) cores derived from 379 patients. Patients were categorized according to low (<1.56, N = 141), intermediate (1.56-1.75, N = 148), and high (>1.75, N = 90) fractal dimension. Cox proportional-hazards regression was used to assess the relationship between disease-specific and overall survival and fractal dimension, tumour size, grade, nodal status, estrogen receptor status, and HER-2/*neu *status.

**Results:**

Patients with higher fractal score had significantly lower disease-specific 10-year survival (25.0%, 56.4%, and 69.4% for high, intermediate, and low fractal dimension, respectively, p < 0.001). Overall 10-year survival showed a similar association. Fractal dimension, nodal status, and grade were the only significant (P < 0.05) independent predictors for both disease-specific and overall survival. Among all of the prognosticators, the fractal dimension hazard ratio for disease-specific survival, 2.6 (95% confidence interval (CI) = 1.4,4.8; P = 0.002), was second only to the slightly higher hazard ratio of 3.1 (95% CI = 1.9,5.1; P < 0.001) for nodal status. As for overall survival, fractal dimension had the highest hazard ratio, 2.7 (95% CI = 1.6,4.7); P < 0.001). Split-sample cross-validation analysis suggests these results are generalizable.

**Conclusion:**

Except for nodal status, morphologic complexity of breast epithelium as measured quantitatively by fractal dimension was more strongly and significantly associated with disease-specific and overall survival than standard prognosticators.

## Background

The prognostic assessment of breast cancer is based on factors that determine a patient's relapse risk, and together with predictive factors (e.g., estrogen-receptor status), it is used to make optimal therapeutic decisions regarding adjuvant systemic therapy [1]. Such decisions provide a balance between the potential benefit and associated costs and side effects of treatment [1]. Therefore, it is necessary to have sensitive and specific prognosticators to accurately define risk category for breast cancer.

Currently, the most significant prognosticator for women with breast cancer is axillary lymph node status [1-4]. For node-positive patients, there is a direct relationship between the number of involved axillary nodes and the risk for distant recurrence [4]. However, despite the usefulness of lymph node status, recommendations for systemic adjuvant chemotherapy are not entirely straightforward. For example, five-year survival rates show that approximately 15% of all node-negative patients with larger tumor sizes (>1 cm) may benefit from systemic adjuvant therapy, but about 85% would survive without it [5]. Furthermore, approximately one-third of node-positive patients are free of recurrence after local-regional therapy [6-8].

Other major prognostic risk factors, especially for node-negative patients, are tumor size and histological tumor grade [1-4,9,10]. For node-negative patients, tumor size is a powerful prognostic factor that is used routinely to make adjuvant treatment decisions [6,11], and tumor grade is primarily used to make decisions for cases in which the tumor sizes are borderline [1,2,5]. Although tumor grade has prognostic value, significant inter-observer variation in grading still exists [12-14]. as pathologists are assessing complex histological characteristics in a semi-quantitative manner.

It is known that invasive breast cancer (a malignant neoplasm) demonstrates partial or complete lack of structural organization and functional coordination with surrounding normal tissue [15]. The idea central to this study is that this loss of structural organization and functional coordination manifests itself in the form of an increase in morphologic complexity of the epithelial components at the sub-cellular, cellular, and multi-cellular levels, and the degree of this complexity can be quantified and related to patient outcome. A method that lends itself particularly useful for quantitatively characterizing complex pathological structures at different scales, is based on fractal analysis [16,17]. In this study, we assess the prognostic value of a recently developed novel technique [18] to measure the fractal dimension of segmented histological structures of breast tissue microarray (TMA) cores stained with pan-cytokeratin to highlight the morphology of epithelial architecture.

## Methods

### Patient Characteristics

A total of 408 patients with primary invasive ductal carcinoma (IDC) of the breast were selected retrospectively from the Calgary Regional Hospitals after appropriate ethics approval from the Institutional Review Board (IRB). It should be noted that the IRB did not require patient consent for this study as it was a retrospective study in which many of the patients were deceased and the risk of exposing patient confidentiality was extremely low. Of these, 379 patients had at least one of three TMA cores that was sufficiently stained for fractal analysis. The age range of these patients at diagnosis was 34 to 95 with a mean and median age of 65 and 66, respectfully. Stage information was available for 375 of 379 patients with the following frequency distribution: 225 (60.0%) patients were Stage I, 99 (26.4%) were Stage II, and 51 (13.6%) were Stage III. All patients selected had received adjuvant tamoxifen treatment between 1988 and 2006. Cases were identified with Alberta Cancer Board records of patients who had received tamoxifen treatment without chemotherapy. In summary, the inclusion criterion was any patient who had adequate tissue for TMA construction, and had received adjuvant tamoxifen treatment but no adjuvant chemotherapy.

### Sample Preparation

Whole sections stained with Hemotoxylin and Eosin (H&E) were used to select tumor areas for the TMA cores. Fourteen breast TMA blocks containing an average of 94 tissue cores were constructed from formalin-fixed, paraffin-embedded, previously untreated breast cancer tissue. To ensure there was no selection bias, three 0.6 mm cores were chosen randomly from cancerous areas of each donor block to construct the recipient TMA core block, and the Leica RM2235 microtome (Leica Microsystems Inc.) was used to cut 4 μm thick sections from each TMA donor block. In a previous study with prostate cancer specimens, we showed that fractal analyses of specimens stained with pan-cytokeratin provide greater classification performance (benign versus high grade) than serial sections of the same specimens stained with H&E [18]. The reason for this is that pan-cytokeratin isolates and highlights the morphology of epithelial components and excludes structures that do express pathological relevance in the form of morphologic complexity (i.e., connective tissue components). Hence, we stained all the TMA sections with pan-cytokeratin. This staining was performed using Ventana Benchmark LT. Protease 1 antigen retrieval was used followed by Ventana pre-diluted pan-cytokeratin (cat. No. 760-2135) antibody with an incubation time of 32 minutes. A Ventana ultraview™ DAB detection system was used for detection.

### Image Acquisition of TMA Cores

Microscopic images of the TMA cores were acquired with an AxioCam HR digital camera (Carl Zeiss, Inc.) mounted on an optical microscope (Zeiss Axioscope) at a magnification of 10 × objective. The AxioCam HR has pixels of size 6.7 *μm *× 6.7 *μm*, which are 1.06 *μm *× 1.06 *μm *in apparent size at the combined magnifications of 10 × objective and 0.63 × C-mount optical coupling (optical interface between the microscope and digital camera). The images were taken at the camera's native resolution of 1300 × 1030 pixels, and saved in tagged image file format (tif).

### Fractal Analysis to Assess Morphologic Complexity

**Figure 1:**
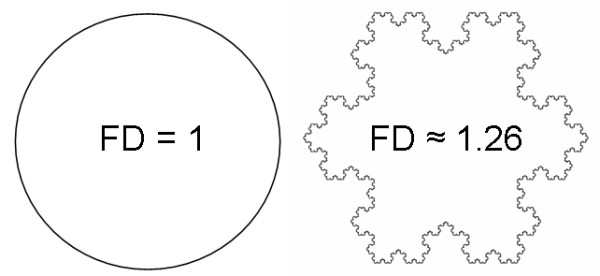
**Both the circle (left) and the Koch snowflake (right) have a topological dimension of 1; however, the fractal dimension (FD) of the Koch snowflake is greater than 1 because it has a more complex morphology than the circle**.

Unlike our intuitive notion of dimension (i.e., topological dimension), fractal dimension can be a non-integer value, and the greater the morphologic complexity of an object, the higher its fractal dimension relative to its topological dimension (Figure 1). Fractal dimension quantifies the level of structural complexity by assessing the variation in the level of detail in a structure as the structure is examined at different scales [19]. Hence, it lends itself naturally to characterizing irregular structures that maintain a constant level of complexity over a range of scales.

**Figure 2:**
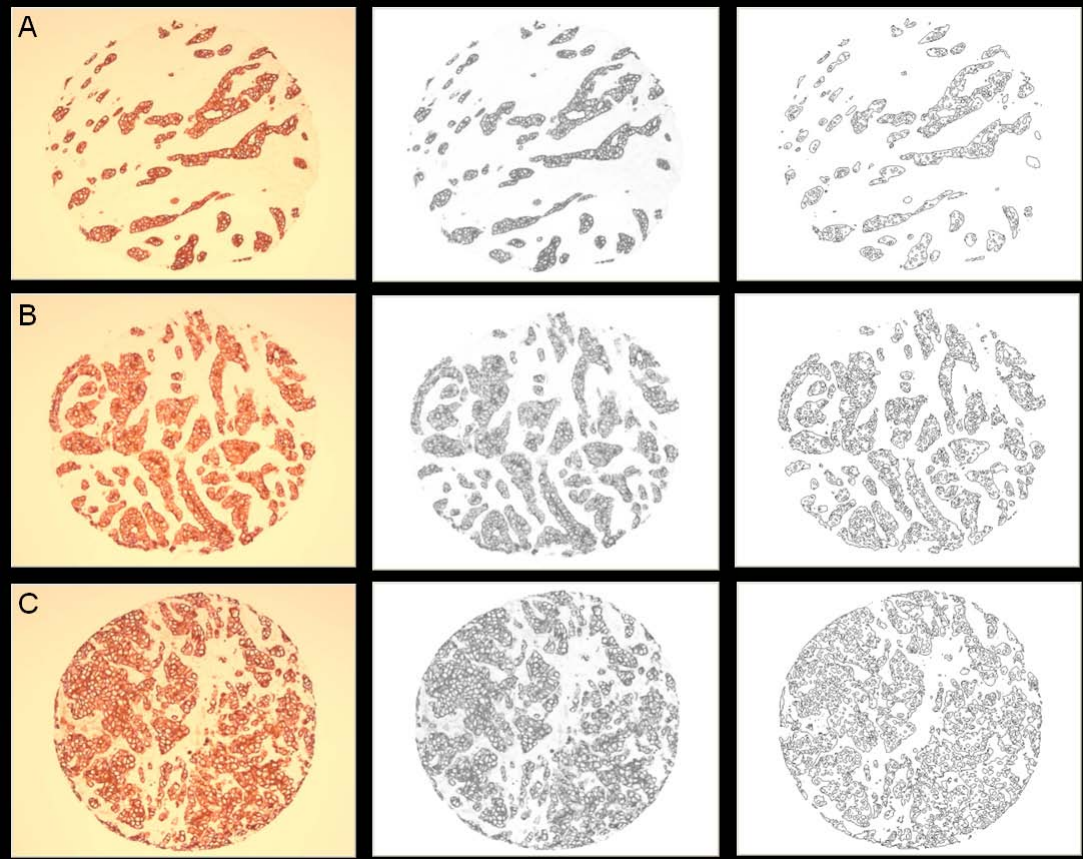
**Pan-keratin stained TMA cores (left column) representative of A: low (< 1.56), B: intermediate (1.56-1.75), and C: high (> 1.75) fractal dimension categories, the corresponding background corrected gray-scale images (center column), and the corresponding outline morphology images (right column) from which fractal dimensions are computed**.

**Figure 3:**
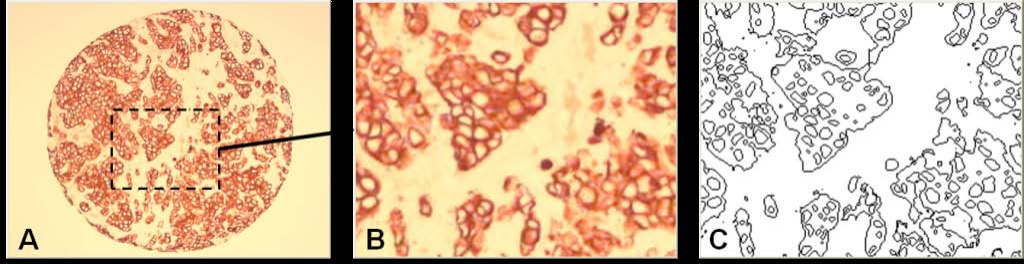
A: Original image (Figure 2C); B: Magnified portion of A, the dashed rectangular region; C: Segmented outline structures corresponding to the magnified image region.

In this study, we applied an automated fractal analysis technique we developed in previous work [18] to quantify the morphologic complexity of breast epithelium, a pathologically relevant histological feature. In summary, this technique involves the following steps:

1. Application of a histological stain to tissue specimens in order to highlight and isolate the histological structures of interest. In this case, these structures include the outlines of the epithelial components comprising the multi-cellular structures (gland formations), cellular structures (individual cell shapes), and sub-cellular structures (distribution of keratin within the cells and nuclear shape).

2. Image acquisition and background correction of stained specimens. The background correction was done by acquiring a "blank" image (under the same imaging conditions used to acquire the TMA images), and using this "blank" image to subtract the non-uniform background luminance [18]. The resulting background corrected images are converted to grey-scale (Figure 2).

3. Application of a series of intensity thresholds to convert the grey-scale version of the image specimen into a series of binary images from which histological morphology outlines are derived (Figure 2). Figure 3 shows a sample magnified region of Figure 2A to illustrate the segmented morphology outlines in more detail.

4. Application of the box counting method [19] (with appropriate spatial scale range - 10 to 50 μm) [20] to compute the fractal dimension of each outline image obtained from step 3.

5. Identification of the global maximum from a plot of fractal dimension versus intensity threshold. This maximum corresponds to the fractal dimension of the pathological morphology.

In previous work, we showed that our method of finding the fractal dimension is independent of changes in microscope illumination setting or stain uniformity and intensity [18]. Also, it should be noted that fractal dimension is not affected by magnification as long as the field of view of the specimen image still contains the scale range of the structures of interest over which the fractal dimension was found to be constant.

Our automated fractal analysis method was applied to a total of 1224 TMA cores (3 cores for each of the 408 patient samples). For each patient, the TMA core with the maximum fractal dimension was used for the statistical analysis in this study. The rationale for choosing the maximum fractal dimension from the sampled tissue cores is to reduce the possibility that the other TMA cores from a given patient contain only benign or more highly differentiated tissue. That is, it is expected that the TMA core with the maximum fractal dimension is representative of the malignant neoplasm that has deviated most from normal cellular/glandular breast morphology, and therefore it is the most probable indicator of abnormal and/or aggressive tumor growth with metastatic potential.

For 379 of the 408 patients (92.9%), fractal dimension was successfully measured in at least one of the three TMA cores generated per patient, and it could not be determined for the remaining 29 patient specimens due to insufficient staining (i.e., less than half of the specimen being stained) or specimen folding. Eight of the 29 patients could not be assessed because all 3 of their TMA cores resulted in a "blank" slide. The breakdown of the number of patients for which the TMA cores were sufficiently stained for fractal analysis was as follows: 36 patients (9.5%) had one evaluable core, 105 patients (27.7%) had two evaluable cores, and 238 patients (62.8%) had three evaluable cores.

### Statistical Analyses

For purposes of analyses, it is often useful to convert a measured variable to a categorical variable so as to place patients into graded risk strata. As the particular fractal analysis technique we developed is novel, there are no established cutpoints available. Although several methods exist to determine cutpoints, namely biological determination, data-oriented, and outcome-oriented, there is no single method or criterion to specify which approach is best. For the present analyses, we used a data-oriented approach to select two cutpoints. The first cutpoint was chosen to correspond to the upper quartile (75^th ^percentile) of the fractal dimension data, and the second cutpoint was chosen as the median of the remaining lower three-quarters of the data. Two cutpoints, rather than one, were chosen to assess whether there was a graded relationship between fractal dimension and patient prognosis.

Associations between categorized fractal dimension scores and clinicopathological variables were assessed for statistical significance using a chi-square test. Kaplan-Meier methods were used to estimate 10-year disease-specific and overall survival rates and the logrank test was used to compare the curves for statistical significance. Disease-specific survival was measured from the date of diagnosis to the date of death from cancer or date of last follow-up. Overall survival was measured from the date of diagnosis to the date of death from any cause or date of last follow-up. The above analyses were repeated using Cox proportional hazards regression modeling to assess whether any of the clinicopathological variables influenced the findings. The proportionality assumption was assessed for all covariates using Log-Minus-Log Survival Plots and none violated the assumption. Statistical analyses were performed using SAS 9.2 software (SAS Institute Inc).

The prognostic accuracy of fractal dimension in predicting death from breast cancer and death from any cause was quantified by the area under the curve (AUC) from a receiver operating characteristic (ROC) analysis. Values of AUC range from 0.5 (chance accuracy) to 1.0 (perfect accuracy), with the following intermediate benchmarks: 0.6 (fair), 0.7 (good), 0.8 (excellent), and 0.9 (almost perfect). For the analysis, the predicted probability of outcome from a Cox regression model was considered as a continuum. The actual occurrence of outcome was used as the comparative standard.

A split-sample cross-validation was performed to assess the generalizability of the results [21]. The process consisted of splitting the original sample of 379 patients into a training set of 190 patients and a validation set of 189 patients using random sampling. A regression equation was derived in the training set and the AUC between the observed and predicted response values was calculated. The regression coefficients from the training set were then used to calculate predicted values in the validation set. The AUC between these predicted values and observed values in the validation set was calculated, and is called the cross-validation coefficient. The shrinkage coefficient was calculated as the difference between the AUCs of the training and validation sets. The smaller the shrinkage coefficient, the more confidence one can have in the generalizability of the results. Although there are no clear guidelines regarding the magnitude of shrinkage, except that smaller is better, values less than 0.10 indicate a generalizable model. Given a satisfactory shrinkage coefficient, the data were combined from both sets and a final regression equation was derived based upon the entire sample.

Out of 379 evaluable patients, several had missing data: 15 (9.0%) tumor grades, 4 (1.1%) lymph node status, 15 (4.0%) estrogen-receptor status, and 12 (3.2%) HER-2/neu status. Rather than excluding these patients from the analyses and reducing the sample size, missing data were imputed using the predicted mean approach in SOLAS 3.0 software (Statistical Solutions, Ltd.). Imputation bias was assessed by re-running all the analyses and excluding any patient with missing data. As the estimates were similar, the results are reported with the imputed data.

## Results

### Fractal Analysis of the TMA Cores

Fractal dimension scores ranged from 1.08 to 1.97, with a median of 1.62, lower quartile 1.49, and upper quartile 1.75. There was moderate level of relatedness (intraclass correlation = 0.51) among the cores. Using the data-oriented approach to select two cutpoints, fractal dimension values < 1.56 were considered low (N = 141), 1.56-1.75 as intermediate (N = 148), and > 1.75 as high (N = 90). Figure 2 shows representative TMA cores from these fractal dimension categories. One can see from this figure that the classification of TMA cores into low, intermediate, and high fractal dimension categories (A-C) corresponds to the increasing complexity of outline morphology.

### Relationship between Fractal Dimension and Standard Prognosticators

The baseline patient characteristics are shown in Table 1. Higher fractal dimension was significantly associated with traditional indicators of poor prognosis, including older age, larger tumour sizes, higher tumour grade, and positive lymph node status. However, fractal dimension was not associated with either estrogen-receptor status or HER-2/neu status.

**Table 1:** Patient Characteristics by Fractal Dimension Category

	Number (%)	< 1.56 (N = 141) % group	1.56 - 1.75 (N = 148) % group	>1.75 (N = 90) % group	P-value
Age					
≤ 55 years	78 (20.6)	23.4	23.7	11.1	0.039
>55 years	301 (79.4)	76.6	76.3	88.9	
Size of tumour					
≤ 2 cm	272 (71.8)	78.7	69.6	64.4	0.047
>2 cm	107 (28.2)	21.3	30.4	35.6	
Grade of tumour					
1 & 2	338 (89.2)	92.9	91.9	78.9	0.001
3	41 (10.8)	7.1	8.1	21.1	
Lymph node status					
Negative	300 (79.2)	85.1	81.8	65.6	0.001
Positive	79 (20.8)	14.9	18.2	34.4	
Estrogen-receptor status					
Positive	355 (93.7)	93.6	93.9	93.3	0.98
Negative	24( 6.3)	6.4	6.1	6.7	
HER-2/neu status					
Negative	350 (92.4)	95.0	89.9	92.2	0.25
Positive	29 (7.6)	5.0	10.1	7.8	

### Fractal Dimension as a Predictor of Outcome

**Figure 4:**
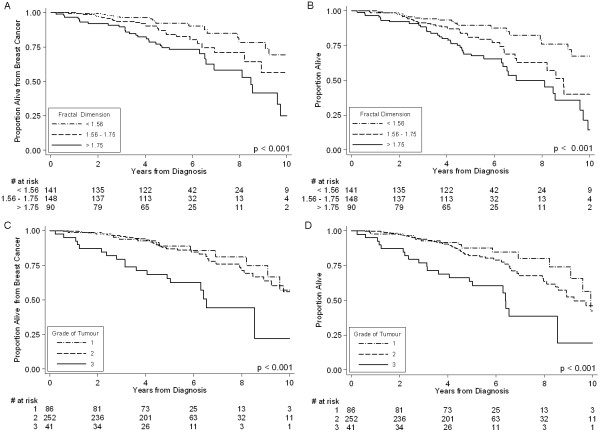
Kaplan-Meier Disease-Specific and Overall Survival Curves by Fractal Dimension Category (Panels A and B, respectively); Kaplan-Meier Disease-Specific Survival and Overall Survival Curves by Tumour Grade (Panels C and D, respectively).

The median patient follow-up was 5.2 years. The 10-year disease-specific and overall survival rates for the entire group of 379 patients were 52.5% and 42.5%, respectively. Patients with higher fractal scores had significantly worse disease-specific survival than those with lower scores (25.0% versus 56.4% versus 69.4%, p < 0.001; Table 2 and Figure 4A). As well, patients with higher scores had significantly worse overall survival (14.2% versus 39.9% versus 67.4%, p < 0.001; Table 2 and Figure 4B). The AUCs for fractal dimension were 0.66 and 0.67 for univariate disease-specific and overall survival, respectively, indicating good levels of prognostic accuracy. As expected, older age, higher grade, and positive lymph node status were significantly predictive of worse outcome, but not the size of the tumour, estrogen-receptor status, or HER-2/*neu *status (Table 2).

**Table 2:** Univariate Results from Kaplan-Meier Analysis and Cox Proportional Hazards Regression

	Number of Patients	10-year Disease- Specific Survival (%)	Univariate Hazard Ratio (95% CI)	P-value	10-year Overall Survival (%)	Univariate Hazard Ratio (95% CI)	P-value
Fractal dimension							
< 1.56	141	69.4	1.0		67.4	1.0	
1.56 - 1.75	148	56.4	1.9 (1.1, 3.6)	0.03	39.9	2.1 (1.2, 3.6)	0.008
>1.75	90	25.0	3.5 (1.9, 6.4)	< 0.001	14.2	3.6 (2.1, 6.1)	< 0.001
Age							
≤ 55 years	78	82.1	1.0		82.1	1.0	
>55 years	301	40.8	3.3 (1.5, 7.2)	0.003	29.1	4.3 (2.0, 9.4)	< 0.001
Size of tumour							
≤ 2 cm	272	49.2	1.0		38.8	1.0	
>2 cm	107	57.0	1.3 (0.8, 2.2)	0.21	47.9	1.3 (0.9, 2.0)	0.18
Grade of tumour							
1 & 2	338	56.1	1.0		45.4	1.0	
3	41	22.1	3.4 (2.0, 5.7)	< 0.001	19.3	2.8 (1.7, 4.6)	< 0.001
Lymph node status							
Negative	300	57.6	1.0		47.8	1.0	
Positive	79	32.2	4.0 (2.5, 6.3)	< 0.001	21.3	3.4 (2.3, 5.1)	< 0.001
Estrogen-receptor status							
Positive	355	53.8	1.0		43.1	1.0	
Negative	24	40.1	1.6 (0.7, 3.4)	0.26	36.0	1.6 (0.8, 3.1)	0.19
HER-2/neu status							
Negative	350	51.6	1.0		42.3	1.0	
Positive	29	60.6	1.2 (0.5, 2.7)	0.71	38.9	1.2 (0.6, 2.5)	0.59

### Tumour Grade as a Predictor of Outcome

Tumour grade was derived from the original pathology reports that included between 10 and 30 board-certified cancer pathologists. In contrast to the distinct separation of the disease-specific survival curves for the different fractal dimension categories (Figure 4A), the disease-specific survival curves for grade 1 and 2 tumours virtually overlaped each other over the entire 10-year follow-up period (Figure 4C). Also, there is virtual overlap in the overall survival curves of tumour grades 1 and 2 for the first 4-year period (Figure 4D). These results suggest that tumour grades 1 and 2 do not discriminate patients with respect to 10-year outcome.

### Multivariate Analysis

Results from Cox proportional hazards regression showed that fractal dimension remained statistically significant even after adjusting for all clinicopathological variables (Table 3). This result implies that fractal dimension is a strong prognostic factor, even though the multivariate hazard ratio (Table 3) is smaller than the univariate hazard ratio (Table 2). The AUCs for the 7-factor regression models were 0.73 and 0.75 for disease-specific and overall survival, respectively. These AUCs increased by only 0.07 and 0.08 when six clinical-pathological factors were added to fractal dimension in the multivariate regression model. The small increase in AUCs incidate that the other clinical-pathological factors contribute little to the prognostic accuracy beyond fractal dimension. It is also worth noting that even with the comparison of grades 1 and 2 as one category versus grade 3 tumours, both disease-specific and overall survival were more strongly and significantly associated with fractal dimension than tumour grade.

**Table 3:** Adjusted Hazard Ratios (95% Confidence Intervals) from Cox Regression

	Death from Breast Cancer	P-value	Death from Any Cause	P-value
Fractal dimension				
< 1.56	1.0		1.0	
1.56 - 1.75	1.9 (1.1, 3.5)	0.043	2.0 (1.2, 3.5)	0.011
>1.75	2.6 (1.4, 4.8)	0.002	2.7 (1.6, 4.7)	< 0.001
Age				
≤ 55 years	1.0		1.0	
>55 years	1.8 (0.8, 4.2)	0.14	2.7 (1.2, 5.9)	0.01
Size of tumour				
≤ 2 cm	1.0		1.0	
>2 cm	1.0 (0.6, 1.6)	0.96	1.0 (0.7, 1.6)	0.88
Grade of tumour				
1 & 2	1.0		1.0	
3	2.1 (1.1, 3.7)	0.01	1.7 (1.1, 3.0)	0.047
Lymph node status				
Negative	1.0		1.0	
Positive	3.1 (1.9, 5.1)	< 0.001	2.6 (1.7, 4.1)	< 0.001
Estrogen-receptor status				
Positive	1.0		1.0	
Negative	1.6 (0.7, 3.7)	0.27	1.5 (0.7, 3.2)	0.26
HER-2/neu status				
Positive	1.0		1.0	
Negative	1.1 (0.5, 2.5)	0.87	1.1 (0.5, 2.4)	0.70

### Split-sample Cross-validation

The generalizability of the aforementioned results was assessed by split-sample cross-validation as described in the statistical analysis section. The results, shown in Table 4 are congruent, not only with each set but also with the results of the entire sample shown in Tables 2 and 3. Specifically, the frequency distribution of low, moderate, and high fractal dimension is similar, as are the 10-year disease-specific and overall survival rates in these three categories. Even with smaller sample sizes, both the training and validation sets still show a pattern of doubling of hazards with higher levels of fractal dimension. The shrinkage coefficients for disease-specific and overall survival were -0.01 and -0.05, respectively, both indicating that fractal dimension is generalizable and that combining data from both sets in the analyses was justified.

**Table 4:** Summary of Split Sample Training Set and Validation Set Results

	Number of Patients	10-year Disease-Specific Survival (%)	Adjusted Hazard Ratio (95% CI)	P-value	10-year Overall Survival (%)	Adjusted Hazard Ratio (95% CI)	P-value
**Training Set Patients**	**190**						
Fractal dimension							
< 1.56	68	69.4	1.0		66.2	1.0	
1.56 - 1.75	76	52.3	2.4 (0.1, 5.8)	0.064	34.2	2.2 (1.0, 4.8)	0.050
>1.75	46	17.0	2.5 (1.0, 6.3)	0.056	16.5	1.8 (0.8, 4.1)	0.17
**Validation Set Patients**	**189**						
Fractal dimension							
< 1.56	73	71.6	1.0		70.6	1.0	
1.56 - 1.75	72	60.5	1.3 (0.6, 3.3)	0.51	44.8	1.7 (0.8, 3.9)	0.18
>1.75	44	32.4	2.3 (1.0, 5.5)	0.06	11.2	3.2 (1.5, 6.9)	0.003

AUC adjusted disease-specific survival analysis, training set = 0.72, validation set = 0.73

AUC adjusted overall survival analysis, training set = 0.68, validation set = 0.73

## Discussion

We previously developed a fractal analysis method to quantitatively measure the morphologic complexity of epithelial architecture [18], and showed a direct association between fractal dimension and breast tumour grade, suggesting that it may be a good surrogate measure of tumour differentiation [22]. In this study we examined the prognostic value of fractal dimension by analyzing 379 specimens from patients with invasive breast cancer, and found that with the exception of nodal status, fractal dimension showed a stronger association with disease-specific survival than standard clinical prognosticators. The potential clinical implications of these results are substantial because to our knowledge, this is the largest and only study of its kind investigating and demonstrating a positive association between the morphologic complexity of breast epithelial architecture (via the fractal dimension metric) and patient outcome. The potential advantages of fractal dimension over conventional tumour grading is that it is a quantitative and reproducible indicator that would be able to provide pathologists with rapid and cost effective high volume analysis from as few as three tissue microarray (TMA) cores per patient.

Ideally, a study investigating the value of a potential prognosticator should only involve patients that have not received any form of adjuvant systemic therapy. However, as noted by Mirza *et al. *[5], such studies are becoming increasingly difficult to perform because systemic therapy is recommended for an ever-widening range of breast cancer patients. Although none of the patients in this study were treated with adjuvant chemotherapy, they were all treated with adjuvant tamoxifen therapy, including the 24 ER-negative patients (note: cases selected for this study where from as far back as 1988 when tamoxifen was occasionally administered to patients with ER-negative tumours). However, even though the patients received a form of adjuvant systemic therapy, the same form of treatment was received by all of the patients leading to the expectation that fractal dimension will be independent of the predictive factor related to tamoxifen therapy (i.e., ER-positive status). Indeed, this appears to be the case, since approximately the same percentage of ER-positive patients are in the low, intermediate, and high fractal dimension groups (Table 1), which likely indicates that tamoxifen therapy has put all of these ER-positive patients on an equal footing. However, another possibility for this result may be that ER status does not affect the morphologic complexity of epithelial architecture. In either case, it may be argued that the use of tamoxifen treated patients in a study investigating the value of a possible prognosticator, although not ideal, does not detract from the ability to assess the prognostic factor's potential relative to other independent prognosticators.

Previous studies have examined the application of fractal analysis for characterizing cancer [23,24] and have shown that fractal dimension can describe the complex pathological structures seen in some cancers; [18,22] however, to our knowledge, our results represent the largest and sole study relating fractal dimension of epithelial architecture to patient outcome. Although we did not use an external patient validation set in this proof of principle study, we employed a data-oriented approach to minimize bias in the selection of cutpoints, as well as, conducting a split-sample cross-validation analysis. This analysis suggests that the results are generalizable, whereby higher fractal dimensions are associated with poorer outcome. This observation demonstrates the high potential of fractal dimension as an image-based prognostic marker, and it is congruent with the notion that malignant breast neoplasms associated with poorer outcome demonstrate partial or complete lack of structural organization and functional coordination with surrounding normal tissue [15]. Furthermore, it implies that changes in the morphologic complexity of architectural components of the neoplasm (i.e., the epithelium) that arise from changes in the functional status of cells in malignant neoplasms can be quantified with fractal analysis.

## Conclusions

In summary, the results of this retrospective study show that fractal dimension is a promising image analysis marker for the prognosis of IDC of the breast. However, its' prognostic value needs to be confirmed in external validation studies, and ultimately in the context of controlled prospective clinical trials. As a step in this direction, in future work, we will investigate the prognostic value of fractal dimension for defining risk category for Stage I (i.e., lymph node-negative and tumour size ≤ 2 cm in maximum diameter), IDC, ER-positive breast cancer patients that have not received any form of adjuvant systemic therapy. Such a study would be especially valuable because in current clinical practice it is still difficult to identify this subgroup of patients that would benefit most from adjuvant chemotherapy. Also, in future work we will investigate the prognostic and predictive value of combining fractal dimension, a morphological index, with a quantitative analysis of mitotic count, which is a cellular proliferation index that has been shown to be a significant prognostic indicator for node-negative breast cancer [5]. These investigations would provide validation of the significance of morphologic complexity of epithelial architecture in node-negative breast cancer, and explore the possible synergy between morphologic complexity and cellular proliferation. Also, they will bring us closer to the realization of an objective prognosticator that can assist clinicians in making optimal treatment decisions regarding adjuvant systemic therapy for invasive breast cancer.

## Abbreviations

AUC: Area under the curve; CI: Confidence interval; ER: Estrogen receptor; FD: Fractal dimension; H&E: Hemotoxylin and eosin; HER-2/*neu*: Human epidermal growth factor receptor 2; IDC: Invasive ductal carcinoma; IRB: Institutional review board; ROC: Receiver operating characteristics; tif: tagged image file format; TMA: Tissue microarray

## Competing interests

With the help of University Technologies International (UTI), the authors are exploring the possibility of commercializing the fractal analysis software used to analyze the breast tissue microarray images in this study.

## Authors' contributions

MT performed the literature search, study design, fractal dimension analysis, and drafted the manuscript and figures. ME participated in the study design, performed the statistical analysis and interpretation, and drafted the statistical analysis and results sections. AM participated in the study design, the generation of the TMA cores and database, and the interpretation of the data. All authors read and approved the final manuscript.

## Authors' information

MT is a board certified Medical Physicist with extensive expertise in radiation oncology physics, and medical imaging and analysis. ME is a distinguished Biostatistician with well over 150 publications, and expertise in the application of statistics to medicine. AMM is a Molecular Pathologist with extensive expertise in breast cancer pathology and the development and clinical implementation of prognostic and predictive molecular biomarkers of cancer.

## References

[B1] Lonning PE Knappskog S Staalesen V Chrisanthar R Lillehaug JR Breast cancer prognostication and prediction in the postgenomic era Ann Oncol 2007 18 1293 1306 10.1093/annonc/mdm013 17317675

[B2] Cianfrocca M Gradishar WJ Controversies in the therapy of early stage breast cancer Oncologist 2005 10 766 779 10.1634/theoncologist.10-10-766 16314287

[B3] Mori I Yang Q Kakudo K Predictive and prognostic markers for invasive breast cancer Pathol Int 2002 52 186 194 10.1046/j.1440-1827.2002.01335.x 11972862

[B4] Cianfrocca M Goldstein LJ Prognostic and predictive factors in early-stage breast cancer Oncologist 2004 9 606 616 10.1634/theoncologist.9-6-606 15561805

[B5] Mirza AN Mirza NQ Vlastos G Singletary SE Prognostic factors in node-negative breast cancer: a review of studies with sample size more than 200 and follow-up more than 5 years Ann Surg 2002 235 10 26 10.1097/00000658-200201000-00003 11753038 PMC1422391

[B6] Elledge RM McGuire WL Prognostic factors and therapeutic decisions in axillary node-negative breast cancer Annu Rev Med 1993 44 201 210 10.1146/annurev.me.44.020193.001221 8476242

[B7] Polychemotherapy for early breast cancer: an overview of the randomised trials. Early Breast Cancer Trialists' Collaborative Group Lancet 1998 352 930 942 10.1016/S0140-6736(98)03301-7 9752815

[B8] Tamoxifen for early breast cancer: an overview of the randomised trials. Early Breast Cancer Trialists' Collaborative Group Lancet 1998 351 1451 1467 10.1016/S0140-6736(97)11423-4 9605801

[B9] Elston CW Ellis IO Pathological prognostic factors in breast cancer. I. The value of histological grade in breast cancer: experience from a large study with long-term follow-up Histopathology 1991 19 403 410 10.1111/j.1365-2559.1991.tb00229.x 1757079

[B10] Henson DE Ries L Freedman LS Carriaga M Relationship among outcome, stage of disease, and histologic grade for 22,616 cases of breast cancer. The basis for a prognostic index Cancer 1991 68 2142 2149 10.1002/1097-0142(19911115)68:10<2142::AID-CNCR2820681010>3.0.CO;2-D 1913453

[B11] Saez RA McGuire WL Clark GM Prognostic factors in breast cancer Semin Surg Oncol 1989 5 102 110 10.1002/ssu.2980050206 2657970

[B12] Meyer JS Alvarez C Milikowski C Olson N Russo I Russo J Breast carcinoma malignancy grading by Bloom-Richardson system vs. proliferation index: reproducibility of grade and advantages of proliferation index Mod Pathol 2005 18 1067 1078 10.1038/modpathol.3800388 15920556

[B13] Robbins P Pinder S de Klerk N Dawkins H Harvey J Sterrett G Histological grading of breast carcinoma: a study of interobserver agreement Hum Pathol 1995 26 873 879 10.1016/0046-8177(95)90010-1 7635449

[B14] Chowdhury N Pai MR Lobo FD Kini H Varghese R Interobserver variation in breast cancer grading: a statistical modeling approach Anal Quant Cytol Histol 2006 28 213 218 16927641

[B15] Rizki A Bissell MJ Homeostasis in the breast: it takes a village Cancer Cell 2004 6 1 2 10.1016/j.ccr.2004.06.019 15261134

[B16] Coffey DS Self-organization, complexity and chaos: the new biology for medicine Nat Med 1998 4 882 885 10.1038/nm0898-882 9701230

[B17] Baish JW Jain RK Cancer, angiogenesis and fractals Nat Med 1998 4 984 10.1038/1952 9734370

[B18] Tambasco M Costello BM Kouznetsov A Yau A Magliocco AM Quantifying the architectural complexity of microscopic images of histology specimens Micron 2009 40 486 494 10.1016/j.micron.2008.12.004 19171487

[B19] Peitgen H Jurgens H Saupe D Chaos and Fractals: New Frontiers of Science 2004 2 New York: Springer-Verlag

[B20] Dixon V Tambasco M Effects of image resolution and noise on estimating the fractal dimension of tissue specimens Anal Quant Cytol Histol 2010 32 269 279 22043503

[B21] Kleinbaum DG Kupper LL Muller KE Nizam A Applied Regression Analysis and Other Multivariable Methods 1998 3 Duxbury Press

[B22] Tambasco M Magliocco AM Relationship between tumor grade and computed architectural complexity in breast cancer specimens Hum Pathol 2008 39 740 746 10.1016/j.humpath.2007.10.001 18439940

[B23] Baish JW Jain KJ Fractals and cancer Cancer Res 2000 60 3683 3688 10919633

[B24] Cross SS Fractals in pathology Journal of Pathology 1997 182 1 8 10.1002/(SICI)1096-9896(199705)182:1<1::AID-PATH808>3.0.CO;2-B 9227334

